# Examining the clinical role and educational preparation of heart failure nurses across Europe. A survey of the Heart Failure Association (HFA) of the European Society of Cardiology (ESC) and the Association of Cardiovascular Nursing and Allied Professions (ACNAP) of the ESC

**DOI:** 10.1002/ejhf.3519

**Published:** 2024-11-08

**Authors:** Loreena Hill, Nicolas Girerd, Teresa Castiello, Tiny Jaarsma, Marco Metra, Giuseppe Rosano, Patrick Savage, Mark J. Schuuring, Maggie Simpson, Izabella Uchmanowicz, Maurizio Volterrani, Rhys Williams, Ekaterini Lambrinou, Camilla Hage

**Affiliations:** ^1^ School of Nursing & Midwifery, Queen's University Belfast UK; ^2^ College of Nursing, Mohammed Bin Rashid University Of Medicine and Health Sciences Dubai United Arab Emirates; ^3^ Université de Lorraine, INSERM, Centre d'Investigations Cliniques 1433, CHRU de Nancy, Inserm 1116 and INI‐CRCT (Cardiovascular and Renal Clinical Trialists) F‐CRIN Network Nancy France; ^4^ Croydon Health Service, Cardiovascular Imaging, Cardiology Department and Kings College London London UK; ^5^ Department of Health, Medicine, and Care Linkoping University Linköping Sweden; ^6^ Institute of Cardiology, ASST Spedali Civili, Department of Medical and Surgical Specialties, Radiological Sciences and Public Health, University of Brescia Brescia Italy; ^7^ Department of Cardiology St George's University Hospital London UK; ^8^ Cardiology Research Fellow, Queens University Belfast Belfast UK; ^9^ Department of Cardiology Medical Spectrum Twente Enschede The Netherlands; ^10^ Department of Biomedical Signals and Systems University of Twente Enschede The Netherlands; ^11^ Queen Elizabeth University Hospital, NHS Greater Glasgow & Clyde Glasgow UK; ^12^ Department of Nursing and Obstetrics Faculty of Health Sciences, Wroclaw Medical University Wroclaw Poland; ^13^ Cardiopulmonary Department IRCCS San Raffaele Roma Rome Italy; ^14^ Exercise Science and Medicine ‐San Raffaele Open University in Rome Rome Italy; ^15^ Princess of Wales Hospital, Cwm Taf Morgannwg University Health Board Wales UK; ^16^ Department of Nursing Cyprus University of Technology Limassol Cyprus; ^17^ Department of Cardiology, Heart Failure GUCH Arrhythmia and Karolinska Institutet Karolinska University Hospital Stockholm Sweden; ^18^ Department of Medicine, Unit of Cardiology Stockholm Sweden

**Keywords:** Heart failure, Disease management, Nurses, Role

## Abstract

**Aims:**

To describe the clinical practice and educational preparation of heart failure (HF) nurses across Europe and determine the key differences between countries.

**Methods and results:**

A survey tool was developed, in English, by the Heart Failure Association Patient Care committee of the European Society of Cardiology (ESC). It was translated into eight languages, before electronically disseminated by nurse ambassadors, presidents of HF national societies and through social media. A total of 837 nurses involved in the daily care of patients with HF from 15 countries completed the survey. Most nurses, 78% (*n* = 395) worked within a hospital outpatient setting, and 51% (*n* = 431) had access to a specialized HF multidisciplinary team. Nurses performed a range of activities including patient education to promote self‐care, virtual and in‐person symptom monitoring. A third had more than 5‐year experience in cardiac care and 22% (*n* = 182) prescribed HF medications. There was a significant correlation between HF nurses that prescribed HF medications and access to a specialist multidisciplinary team (*p* = 0.04). A small number of nurses, mainly from Belgium, supported invasive monitoring (*n* = 68, 8%) with 14% (*n* = 120) of mostly Danish nurses supporting exercise programmes. The majority of nurses surveyed were committed to further academic professional development, with 41% (*n* = 343) having completed a HF course.

**Conclusion:**

The role of the HF nurse varies across Europe, however involvement in patient education, symptom monitoring and follow‐up remain core to their practice. In specific activities including the prescribing of HF medications and involvement in invasive monitoring, practice has advanced with collaboration in the multidisciplinary team. Consequently, harmonization of education, training and career pathways are required to standardize HF care aligned with expert guidelines across Europe.

## Introduction

Heart failure (HF) affects over 65 million individuals globally, with prevalence predicted to rise due to improved survival rates for patients with ischaemic heart disease and an ageing society, posing an enormous pressure on public health systems.^1^, ^2^ The 2021 European Society of Cardiology (ESC) HF guidelines emphasized the role of the multidisciplinary team (MDT) and self‐management strategies to reduce the risk of HF hospitalization and mortality in patients with chronic HF (giving it a Class I, level A recommendation).^3^ Ideally, this MDT should include trained HF specialists from different disciplines (i.e. nursing, pharmacy, physiotherapy), cooperating together in a coordinated approach that transcends primary and secondary settings.^4^ This MDT approach will meet the multifaceted and dynamic needs of individuals affected by HF and their families/caregivers, irrespective of the HF phenotype.^3^, ^5^

Patients with HF often experience a high symptom burden and poor quality of life.^6^, ^7^ For those with HF with reduced ejection fraction (HFrEF), guideline‐directed medical therapy (GDMT) has successfully improved their symptoms, quality of life and extended their lifespan,^8^, ^9^ however it is often underused especially in the elderly.^10^, ^11^ For patients with HF with preserved ejection fraction (HFpEF), care has historically been reserved to optimization of treatment for comorbidities and promote the control of symptoms using diuretics. Recent trials and guidelines emphasize the benefit of sodium–glucose cotransporter 2 inhibitor (SGLT2i) therapy for HF patients across the spectrum of ejection fraction to reduce the composite endpoint of cardiovascular death or hospitalization.^3^, ^12^, ^13^ The optimization of GDMT is an important component of a successful management plan that many HF nurses can support to improve the outcomes of patients with HF.^3^, ^14^, ^15^, ^16^

All healthcare professionals within the MDT play a vital role in promoting the self‐care of patients with HF.^17^ They need to educate and inform patients about their HF condition, teach skills, personalize treatment plans, support individual needs and abilities, as well as engage with patients and their caregivers to make shared decisions regarding ongoing treatment. As digital technology advances, different tools and platforms are being used as a beneficial ‘add‐on’ for patients, caregivers, healthcare professionals and the economy to support long‐term follow‐up.^18^, ^19^, ^20^, ^21^

As the landscape of HF care continues to evolve, insights from current practices of HF nurses are key to shaping new curricula. The Association of Cardiovascular Nursing and Allied Professions (ACNAP) published a core curriculum for cardiovascular nurses and allied professionals in 2023.^22^ The most recent curriculum for HF nurses was published by the Heart Failure Association (HFA) of the ESC in 2016.^23^ Over the last 8 years there has been significant advancements in HF treatment, with a parallel increase in the number of nurses in advanced clinical practice roles.^24^ An update is urgently needed to equip these nurses with the knowledge and skills necessary to meet the challenges of modern HF management. It is timely to evaluate the daily clinical practice and educational preparation of HF nurses within ‘real‐world’ settings to ensure future clinical developments and educational initiatives are appropriate to advance practice. Therefore, the aim of this study was to describe the clinical practice and educational preparation of HF nurses across Europe and determine key differences between countries.

## Methods

### Procedure

The Patient Care Committee of the HFA of the ESC developed a survey tool in 2021. Volunteers from eight different countries translated the English version (online supplementary material) of the survey into their native languages including Spanish, Swedish, Dutch, Italian, German, Cypriot, Lithuanian, and French, ensuring cultural and linguistic adaptation.

Participants were recruited in two phases using a targeted, yet snowball approach.

*Phase One*: Nurse ambassadors in each of the eight countries, as well as the United Kingdom and Ireland received the electronic and translated survey and were asked to share it with key HF nurse colleagues within their networks. *Phase Two*: Presidents of national HF societies received the survey web‐link with a request to distribute it to their membership and to promote participation. Simultaneously, the survey was promoted through social media platforms, including HFA website, Twitter, Facebook and the HF Young Community. This recruitment strategy enhanced reach but made determining the exact target population (number of eligible nurses who received but failed to complete the survey) challenging. Two reminders were sent to promote recruitment.

Ethical approval was obtained from Queen's University, Belfast (United Kingdom) in 2022, with general data protection regulation (GDPR) compliance managed by this institution. The project adhered to the Declaration of Helsinki.^25^

### Tool

The survey tool consisted of 35 questions, allocated into four sections. The sections consisted of questions related to the nurses' scope of daily clinical practice, their clinical experience, training, and education for the role, as well as future educational needs and requirements. Questions were developed and refined by members (i.e. cardiologists and nurses) of the Patient Care Committee, with reference to appropriate EQUATOR (Enhancing the QUAlity and Transparency Of health Research) guidelines.^26^ It was pre‐tested in a small sample of cardiovascular nurses for grammar and understanding. Completion of the survey took a maximum of 10 min.

The survey was disseminated and completed online via an electronic platform (Qualtrics). Participation in the survey was voluntary, with eligible participants accessing the survey via a weblink. Additional information was available about the project, with consent inferred on completion of the survey. To ensure anonymity, IP addresses were not collected. Eligibility criteria required nurses to work at least 50% of their working week with HF patients.

### Statistical analysis

Categorical variables were expressed as frequency and percentages. Continuous data were expressed as mean ± standard deviation or as median and interquartile range where specifically stated. Cross tabulation was used to analyse the relationship between key variables, such as access to multidisciplinary HF team and education, and a chi‐square test was used to assess for difference between groups. A *p*‐value of <0.05 was considered statistically significant. Data were exported to, and analysed using IBM SPSS Statistics V.28.0.

## Results

### Context

**Figure 1:**
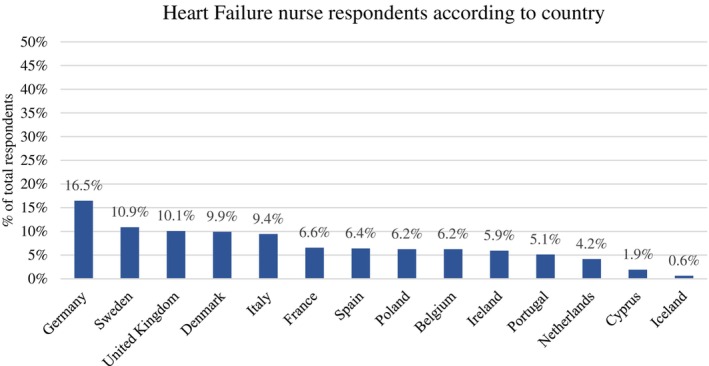
Percentage of heart failure nurse respondents according to country of practice. Percentages expressed as a function of total respondents.

Fifteen ESC countries were represented in the sample of 837 nurses (*Figure* *1*).

The majority of nurses were female (*n* = 643, 85%), aged over 35 years old (*n* = 562, 75%), and working within a hospital setting (*n* = 395, 78%). One third of the HF nurses had more than 5‐year experience in cardiac care (*n* = 282, 34%). Many of the nurses' practice involved assessing patients with HF in both inpatient and outpatient departments (*n* = 212, 42%). Only 34 nurses (7%) worked solely within a community setting (*Table* *1*).

**Table 1:** Demographic and work characteristics of the sample

Variable	Frequencies
Age (*n* = 754)	
<35 years	192 (25%)
>35 years	562 (75%)
Reported sex (*n* = 754)	
Female	643 (85%)
Work setting (*n* = 509)	
Community	34 (7%)
Hospital	395 (78%)
Integrated hospital and community	80 (15%)
Hours per week in direct contact with patients with HF (*n* = 563)	
0–10 h	138 (25%)
11–20 h	162 (29%)
21–30 h	104 (18%)
>31 h	159 (28%)
Education (*n* = 450)	
Diploma or certified course	132 (29%)
Bachelor's degree	123 (27%)
Master's degree	129 (29%)
Doctorate	23 (5%)
Other	43 (10%)
Additional HF training post‐qualification (*n* = 457)	
Yes	305 (67%)
No	152 (33%)

HF, heart failure.

When asked ‘which patients do you routinely assess, treat and care for’, more nurses reported patients with HFrEF (*n* = 480), followed by patients with HF with mildly reduced ejection fraction (HFmrEF, *n* = 355), with a lower number reporting patients with HFpEF (*n* = 311). Nearly half of the nurses surveyed (41%, *n* = 368) assessed more than six patients with advanced HF or had New York Heart Association class III/IV symptoms every month.

Approximately half of the nurses were members of a MDT (*n* = 431, 51%), alongside cardiologists (*n* = 384, 45%), physiotherapists (*n* = 183, 22%), or dieticians (*n* = 157, 19%). General practitioners were mentioned by 90 nurses (11%), as being members of their HF MDT (online supplementary *Table* *S1*).

### Clinical practice

Questions asked and subsequent analysis on the nurses' daily practice were in accordance with activities specified within the ESC HF guidelines.^3^, ^27^ Three main topics were identified, including optimization of pharmaceutical treatments, daily activities, clinical investigations requested, and patient‐reported outcome measures (PROMs).

#### Optimizing pharmacological therapies

**Figure 2:**
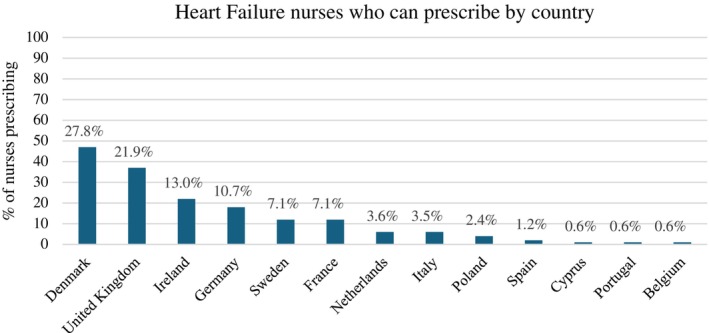
Percentage of heart failure nurses who prescribe guideline‐directed medical therapy according to country of practice. Percentages expressed as a function of total respondents.

**Figure 3:**
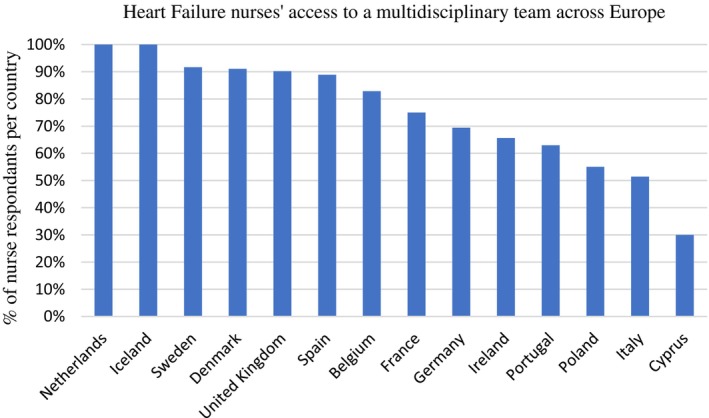
Heart failure nurses' access to members of a heart failure multidisciplinary team according to their country of practice. Data were available for 473 respondents This graph shows yes/no responses by country with percentages expressed as a function of the total respondents from each country.

In total, 22% (*n* = 182) of the nurses reported that they could prescribe or adjust HF medications either independently (*n* = 80, 44%) or according to a protocol (*n* = 102, 56%). However this practice varied according to country of residence and access to MDT (*Figures* *2* and *3*). For example, Danish and British nurses are more likely to prescribe, with Italian, Cypriot and Polish nurses having notably lower rates of prescribing and access to the MDT. Respondents that could prescribe HF medication were noted to be more likely to have access to a specialist HF MDT (42% vs. 31.7%, *p* = 0.04).

All four pillars of GDMT were routinely prescribed or adjusted by HF nurses. In addition, loop (*n* = 177) and thiazide (*n* = 107) diuretics were prescribed orally. Within primary care, only a small number of nurses (*n* = 99, 21%) were able to administer intravenous diuretics. In many cases, a patient's need for intravenous diuretics warranted a hospital admission (*n* = 284) or referral to emergency department (*n* = 241).

#### Daily activities

**Figure 4:**
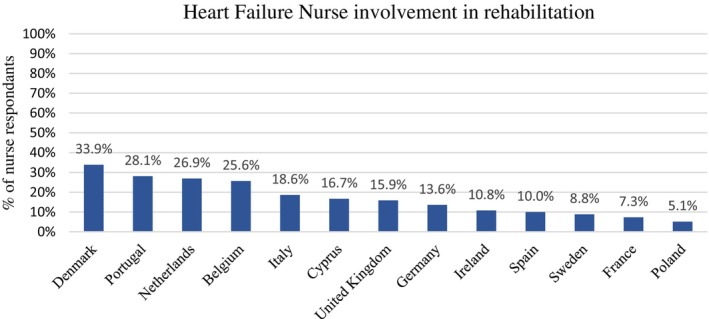
Heart failure nurse involvement in rehabilitation programmes according to country of practice. Percentages are expressed as a function of total respondents.

**Figure 5:**
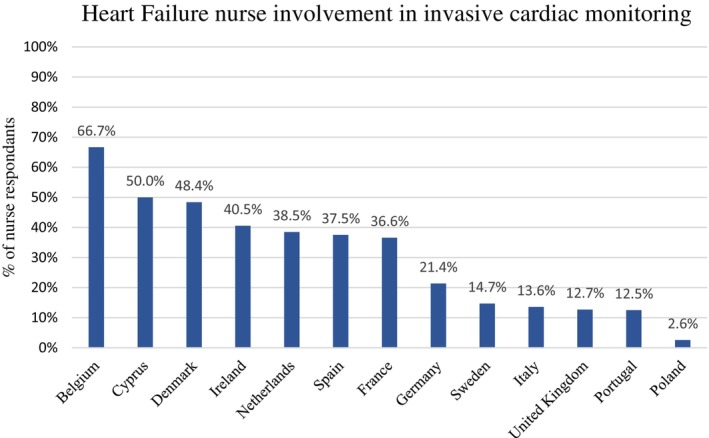
Heart failure nurse involvement in invasive cardiac monitoring, according to country of practice. Percentages are expressed as a function of total respondents.

Patient education was a key role undertaken by many of the nurses, in particular education to promote self‐care (*n* = 465, 56%). Similarly, symptom monitoring was a fundamental component of the role of these nurses, as reported by 55% (*n* = 463) of those who participated in the survey. In addition, remote monitoring of patients with HF was carried out by 24% (*n* = 199) of nurses. The activities HF nurses were least likely to contribute to included invasive telemonitoring (*n* = 69, 8%), undertaken primarily by Belgian nurses, or exercise/rehabilitation programmes (*n* = 120, 14%), supported by third of respondents from Denmark. *Figures* *4* and *5* illustrate the breakdown in the involvement of HF nurses in rehabilitation and invasive monitoring according to country. Data were not collected on the rate of referrals made by the nurses to, for example a rehabilitation programme.

**Figure 6:**
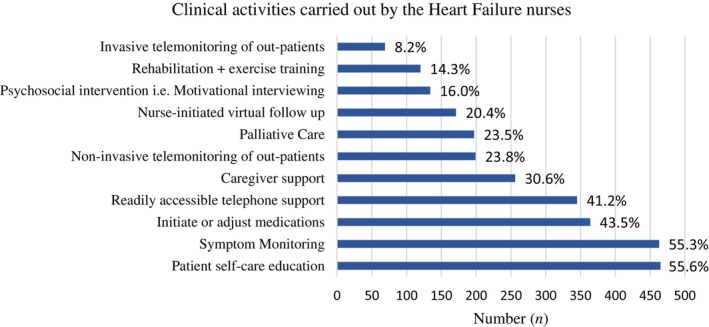
Clinical activities carried out by heart failure nurses. Percentages are expressed as a function of total respondents.

In addition to patient education, HF nurses carried out a range of other activities during their daily practice (*Figure* *6*). These included being an accessible support for patients, via telephone or nurse‐initiated virtual follow‐up. The provision of support for the caregiver was recognized as an important component in the role by nearly one third of HF nurses surveyed (31%, *n* = 256). Only 12 nurses reported the inclusion of a colleague with palliative care expertise within their MDT with 197 (23%) nurses providing palliative care within their day‐to‐day practice.

#### Clinical investigations requested and patient‐reported outcome measures

Main investigations requested by HF nurses were electrocardiograms (*n* = 343, 41%) and routine blood results (*n* = 324, 39%). HF nurses are more likely to be able to request an echocardiogram (*n* = 199, 24%) compared to a chest X‐ray (*n* = 115, 14%) (online supplementary *Figure* *S1*).

**Figure 7:**
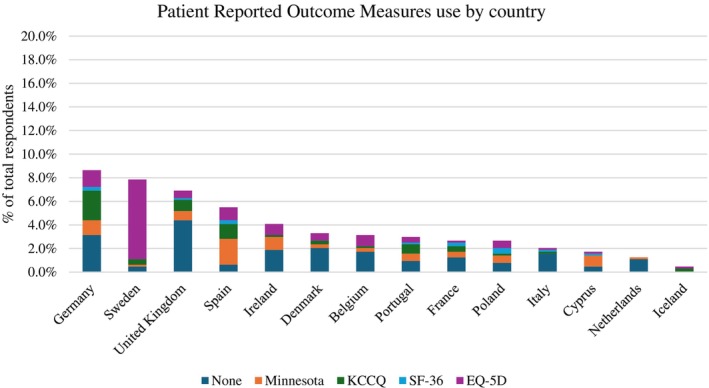
Use of patient‐reported outcome measures by heart failure nurses according to country of practice. EQ‐5D: EuroQol 5‐dimensions; KCCQ, Kansas City Cardiomyopathy Questionnaire; SF‐36: 36‐Item Short‐Form Health Survey.

Some HF nurses routinely undertook a 6‐min walk test to assess the exercise tolerance of their patients (*n* = 150). PROMs were infrequently used (online supplementary  *Figure* *S2*), with 157 nurses reporting that they did not incorporate the use of PROMs within their clinical practice. Those HF nurses who did implement PROMs, the main tools used included EuroQol‐5D (*n* = 98) or Hospital and Depression Scale (HADS) (*n* = 83). *Figure* *7* illustrates the use of PROMs by the nurses according to country. Swedish nurses mainly implemented EuroQol‐5D whilst Spanish nurses used the Minnesota Living with HF questionnaire.

### Experience and education

Over one third of the nurses had more than 5‐year experience in working with cardiac patients (*n* = 282, 34%), with 41% (*n* = 343) indicating they had completed a HF nurse course prior to undertaking their role as a HF nurse. Nurses who disclosed their educational level were educated to diploma level (*n* = 132, 16%), with others to bachelor's degree (*n* = 123, 15%) and master's qualification (*n* = 129, 15%). Of the 446 nurses who disclosed their highest level of education achieved, there was no correlation with education achievement and the ability to prescribe medications (prescribers with diploma *n* = 49, 28%; bachelor's degree *n* = 48, 27%; master's degree *n* = 58, 33%; doctorate *n* = 6, 3.4%).

Less than 30% of nurses (*n* = 255) reported their country had a specific HF course or training. Countries in which training was available included Germany, Sweden, Ireland, United Kingdom (UK), Belgium, Spain and France.

Nurses were invited to document their key educational needs that would support and progress their role as a HF nurse. The main needs expressed included: (i) improved knowledge on GDMT ‘know and optimize evidence‐based medicine’, (ii) advanced clinical knowledge and skills to undertake comprehensive clinical assessment ‘thorough physical examinations to recognize decompensation’. Many nurses also sought (iii) greater awareness of educational opportunities, be that either externally *possibly a continuing education/master's/master's, as most of us have great competencies, which could advantageously be lifted academically* as well as internally *learning from each other (network/collaborate) with hands on learning and be connected to other HF nurses and healthcare workers*.

## Discussion

This study showcased that HF nurses make a valuable contribution to the specialist HF MDT, offering expertise clinically, educationally and in a supportive manner to patients, caregivers and colleagues. Many perform advanced autonomous roles including the independent assessment, prescribing and planning of care for patients, which reflects a development in the role of HF nurses over recent years.^28^ Historically nurses were mainly involved in patient education and in‐person clinical follow‐up, with minimal GDMT titration according to a protocol.^28^ This current survey found HF nurses contribute to invasive monitoring and exercise/rehabilitation programmes, with some being able to prescribe and titrate GDMT independently, as well as assess and follow up patients with HF in‐person and virtually (*Graphical Abstract*). Such findings emphasis the need to provide a contemporary framework and curriculum to equip HF nurses with the necessary expertise through a standardization in the education and training of all HF nurses. A number of countries have developed and published HF nurse competency frameworks, which may account for some of the differences between countries.^29^, ^30^ The new HF nurse curriculum, due to published imminently, will align with the ESC ACNAP Core Curriculum for Cardiovascular Nurses and Allied Professionals^22^ yet also provide a more comprehensive and higher level requirement of independence for nurses caring for patients with HF.

The 2023 ESC focused update of the 2021 HF guidelines gives a Class I, level B recommendation for the initiation and rapid up‐titration of GDMT in the first 6 weeks after discharge for an acute HF hospitalization in accordance with the STRONG‐HF trial.^14^, ^31^ Nurse‐led titration of GDMT can facilitate the optimization of GDMT.^32^, ^33^, ^34^ Within this HF nurse survey, overall only 22% of HF nurses reported that they could prescribe or adjust HF medications independently or according to a protocol. This percentage is notably low despite management of medications recognized in the 2016 HF nurse curriculum.^23^ The proportions of prescribers/non‐prescribers varied according to country and more significantly by access to the HF specialist MDT. This further lends support to the importance of implementing well‐functioning collaborative MDTs and the promotion of training for these nurses, which may help tackle the current clinical problem of therapeutic inertia.^35^

There was reported diversity in practice across Europe in 2006, which remains comparable with results from this current survey. This diversity in practice across countries may reflect not only individual factors in terms of education, but also organizational and country regulations in terms of practice. The prescription of GDMT varied by country, with highest rates of nurse prescribers in Denmark and UK, while Sweden and Germany had higher rates of non‐prescribers to prescribers, despite access to MDTs. The discrepancy may relate to different practices undertaken by the HF nurse within the different countries, for example more focus on invasive monitoring in Belgium and the Netherlands. However, it may also reflect the local regulations where HF nurses can advise on and request initiation of HF treatment and dose adjustments, but the final prescription is signed by the medical practitioner or HF specialist. Thus, the HF nurse is making independent decisions regarding treatment within the collaboration of the MDT but does not ‘prescribe’ in the exact meaning of the word. Furthermore, in a number of countries, such as Italy and Portugal, legislation to enable nurses to prescribe remains lacking.^36^

The two most frequently performed activities by the HF nurses were self‐care education and symptom monitoring, both highly listed as recommended components of a HF management programme. This is supported by previous evidence from semi‐structured interviews whereby HF nurses identified patient education and self‐care support as one of their main tasks, compared to general practitioners and cardiologists who reported that they provide little education.^37^ The importance of patient education to promote self‐care can often be underestimated, however it has been shown to improve patients' quality of life and reduce their hospitalizations.^17^, ^38^, ^39^ Also frequently reported by the HF nurses was their readily available presence, be that via telephone and/or virtually, to offer support, information and in some cases immediate in‐person assessment to prevent worsening of HF symptoms, thus avoidance of a hospital admission. In the REWOLUTION surveys, performed worldwide in patients with HF, 82% reported they took their medication as prescribed, however >70% wanted more information about HF, its consequences, prognosis, and treatments. This information was preferably delivered by healthcare professionals and 44% of patients wanted more frequent appointments with an HF nurse.^40^

Many of the HF nurses work within a hospital outpatient setting, with evidence indicating that nurse‐led HF clinics within primary care reduce the rate of hospitalization and improve patients' quality of life.^21^, ^41^, ^42^ An ‘outreach’ or transitional approach to support the recently discharged patient is that of remote monitoring.^43^ Strategies, populations, and settings have in many trials differed and not surprisingly the results. In a recent meta‐analysis of 61 studies, a trend towards a reduction in deaths and hospitalizations is suggested (1‐year mortality odds ratio 0.54, 95% confidence interval 0.39–0.76).^44^ In the post‐pandemic era, there has been a transition to increase the implementation of remote monitoring, possibly stimulated by patients' receptiveness and participation.^45^, ^46^, ^47^ Invasive telemonitoring was only reported by 69 (8%) of the HF nurses in our survey. With novel tools providing practical information, this is an area where nurses can more easily monitor patients at home facilitating early detection of potential adverse events.^48^ Indeed there is a need for novel and creative implementation whereby HF team structure and member roles such as the HF nurse, fully embrace remote monitoring and telemonitoring to pave the way for success and optimal patient care.^49^

### Limitations

Results from this survey detail the current practice and educational preparation of HF nurses across 15 ESC countries, with identification of disparity across countries with an urgent need for harmonization. As HF centres apply for accreditation and recognition (iCARE‐HF), these results should inform targeted strategies to meet the educational needs of HF nurses. The study does have some limitations. The survey tool was developed and refined by HF experts; however additional evaluation may improve the reliability of results. The dissemination strategy led to an inability to determine how many nurses received the invitation to participate, therefore unable to calculate response rate. Answers were self‐reported, with the possibility of response bias and questions being misinterpreted. Therefore, further research is needed to examine the organizational and country specific challenges to prioritize HF nurses and improve parity in education accreditation.

## Conclusion

This survey has contributed to a greater understanding of the role of the HF nurse in contemporary HF management across Europe. Optimizing HF therapies, empowering patients with self‐care strategies and symptom assessment remain the key components of the role. This is underpinned by experience in cardiology and academic performance. Having access and being recognized as a key member within the multidisciplinary HF team empowers HF nurses to broaden their scope of practice, ultimately benefiting both patients and healthcare services.

The 2016 HF nurse curriculum aimed to standardize the knowledge, skills and behaviours of the HF nurse.^23^ With the demand for rapid up‐titration of GDMT, increased clinical and educational activities undertaken by HF nurses, it is timely to review and update the HF nurse curriculum to promote their advanced training and educational pathways, through the provision of a framework fit for the modern HF nurse.^50^ Furthermore, projects such as iCARE‐HFA promote the importance of accredited educational programmes for HF nurses across Europe.^51^ Evaluating the role of HF nurse and identifying commonalities as well as differences in practice and education, helps inform opportunities to invest professional and HF service development. Ultimately, with the aim to enhance patient outcomes and the standard of HF care delivered by the HF nurses across healthcare systems and Europe.

## Implications for education and practice

1. Development of a European HF nurse career pathway that aims to reduce the diversity across countries and standard practice.
2. Update of the HF nurse curriculum to support professional development and optimize patient care.
3. Recognize and support the important role HF nurses play in the implementation of GDMT and education to patients to promote self‐care.
4. Consideration should be given for the development and implementation of a programme, similar to the certification for HF physicians, for nurses within the ESC community (ACNAP and HFA).
5. HF nurses require accreditation for their educational and clinical advancement.

## Supporting information


**Appendix S1.** Supporting Information.
