# Supplementary material for: Examining the clinical role and educational preparation of heart failure nurses across Europe. A survey of the Heart Failure Association (HFA) of the European Society of Cardiology (ESC) and the Association of Cardiovascular Nursing and Allied Professions (ACNAP) of the ESC
Source: Eur J Heart Fail. 2024 Nov 8;27(2):388–97. doi: 10.1002/ejhf.3519 (PMC11860739; doi:10.1002/ejhf.3519)
Supplement: Supplementary file 1 — Appendix S1. Supporting Information. [file EJHF-27-388-s001.docx]

**Supplementary File 2**

**Figure 8:** Cardiac investigations requested by Heart Failure nurses

Bar chart demonstrating number of HFN’s from questionnaire responses and which cardiac investigations they have the facility to request. Amongst theses respondents, 99 (13.1%) where unable to request any tests and 41 (5.4%) could select tests defined as ‘other’, including ambulatory BP monitoring, cardiac imaging and exercise stress testing.

**Figure 9**: Patient reported outcome utilised by Heart Failure nurses

Bar chart demonstrating various patient reported outcome tools utilised by HFN survey respondents. 6-MWT: Six-minute walk test, EQ-5D: Choice EuroQoL-5D, HADS: Hospital Anxiety and Depression scale, MLwHFQ: Minnesota Living with Heart Failure questionnaire, EHFScBs: European HF Self-care behaviour scale, KCCQ: Kansas City Cardiomyopathy questionnaire, MMSE: Mini Mental State Examination, SF-36: Short-Form 36-item health survey, MoCA: Montreal cognitive assessment.

Table 2: Number of nurses who have access to a Multidisciplinary team according to country

| **Do you have access to a Heart Failure Multidisciplinary team?** | | |
| --- | --- | --- |
| **Country** | **Yes** | **No** |
| Sweden | 55 | 5 |
| Denmark | 51 | 5 |
| Germany | 50 | 22 |
| United Kingdom | 46 | 5 |
| Belgium | 29 | 6 |
| Spain | 24 | 3 |
| Ireland | 21 | 11 |
| France | 18 | 6 |
| Italy | 18 | 17 |
| Netherlands | 17 | 0 |
| Portugal | 17 | 10 |
| Poland | 11 | 9 |
| Iceland | 3 | 7 |
| Total | 360 | 106 |
